# From microtubule remodeling to clinical translation: the multifaceted roles of vasohibin-1 in disease modulation

**DOI:** 10.3389/fimmu.2026.1759658

**Published:** 2026-02-09

**Authors:** Keke Wei, Hui Li, Huiquan Huang, Yinyan Ban, Xiangbin Luo, Chaoren Zhang, Jian Guo, Minghua Tan, Minzhen Qin

**Affiliations:** 1 Department of Critical Care Medicine (Ward 1), Baise People’s Hospital, Affiliated Southwest Hospital of Youjiang Medical University for Nationalities, Baise, Guangxi, China; 2 Division 1, Department of General Surgery, People’s Hospital of Longlin Various Nationalities Autonomous County, Baise, Guangxi, China; 3 Department of Internal Medicine (Unit 1), People’s Hospital of Longlin Various Nationalities Autonomous County, Baise, Guangxi, China; 4 Department of Gastroenterology, Baise People’s Hospital, Affiliated Southwest Hospital of Youjiang Medical University for Nationalities, Baise, Guangxi, China

**Keywords:** angiogenesis regulation, functional paradox, microtubule carboxypeptidase, targeted therapy, vasohibin-1

## Abstract

Vasohibin-1 (VASH-1) is an endothelial protein that serves as a negative feedback regulator of angiogenesis. Through its microtubule carboxypeptidase activity, VASH-1 plays a key role in vascular homeostasis. While previous studies have investigated its involvement in vascular regulation, most have focused on isolated functions or specific disease models, without systematically addressing its multi-dimensional regulatory mechanisms, tissue-specific functional paradoxes, and translational potential. This review provides a comprehensive analysis of VASH-1’s biological characteristics: its expression is induced by pro-angiogenic factors, and it forms a functional complex with SVBP. In pathological contexts, VASH-1 exhibits paradoxical expression patterns—downregulated in neuroendocrine tumors but upregulated in bladder cancer—and demonstrates tissue-specific functions that either inhibit or promote angiogenesis. Clinically, VASH-1 serves both as a diagnostic marker (e.g., serum biomarker and tissue-based prognostic indicator) and a therapeutic target (such as improving renal disease or promoting tumor vascular normalization). This review aims to elucidate the complex physiological and pathological roles of VASH-1, providing a foundation for future precision intervention strategies targeting VASH-1 in disease diagnosis and therapy.

## Graphical Abstract

**Figure:**
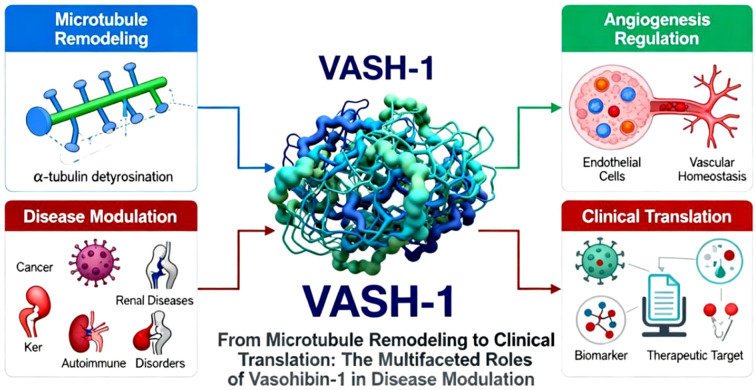

## 1 Introduction

The dynamic equilibrium of angiogenesis is maintained through the precise orchestration of endogenous regulatory networks. Vasohibin-1 (VASH-1), an autocrine negative feedback regulator secreted by endothelial cells, has become a central focus in vascular biology due to its unique functional triad: angiogenesis inhibition, microtubule remodeling, and fibrosis regulation. Its expression is controlled by a multi-tiered regulatory network: VEGF induces transcription *via* the VEGFR2/PKCδ/ERK axis (1, 2), while miRNAs such as miR-10a/b suppress translation (3). Functionally dependent on heterodimerization with SVBP, VASH-1 mediates α-tubulin detyrosination through its microtubule carboxypeptidase activity (4, 5), thereby modulating cytoskeletal stability and angiogenic signal transduction (6).

Beyond classical vascular regulation, VASH-1 is involved in a range of physiological processes, including vascular maturation, where it is enriched in the termination zones of nascent vessels to promote stabilization (7), multi-organ protection by inhibiting the TGF-β/Smad3 pathway to mitigate renal fibrosis (8), and immune microenvironment remodeling through suppression of NF-κB signaling to reduce inflammatory infiltration (9). Notably, VASH-1 exhibits tissue-specific functional paradoxes, such as pro-angiogenic activity in diabetic tissues (10) and dynamic responsiveness to vascular demands in skeletal muscle (11).

Studies on disease association reveal paradoxical expression patterns: systemic downregulation in neuroendocrine tumors (12) contrasts with a positive correlation with aggressiveness in bladder cancer (13). In colorectal cancer (CRC), spatial heterogeneity between tumor cells and stroma leads to functional reversal (14, 15). The clinical translational value of VASH-1 is further highlighted by its role as a serum biomarker for pre-eclampsia risk assessment (16), adenovirus-delivered therapy for ameliorating diabetic nephropathy (DN) (17), and spatiotemporally controlled vascular normalization achieved through sonoporation (18).

This review explores the molecular regulatory network of VASH-1, its disease-specific functional reprogramming, and the challenges to clinical translation, with a focus on organ-targeted delivery systems and multi-omics-integrated strategies to elucidate its microenvironment-dependent functional plasticity.

## 2 Biological characteristics of vasohibin-1

### 2.1 Molecular origin and basic features

VASH-1 is an endogenous protein encoded by a gene located on human chromosome 14q24.3 (19). The gene spans approximately 13 kb of genomic DNA, consisting of eight exons, and transcribes a 5,589-base-pair mRNA, which encodes a 365-amino-acid protein with a molecular weight of approximately 44 kDa (20). VASH-1 expression is tightly regulated at multiple levels: Transcriptional regulation involves positive regulators like VEGF through the VEGFR2/PKCδ/ERK axis (1, 2, 21) and lncRNA H19, which counteracts EZH2-mediated promoter methylation (22). In contrast, negative regulation is exerted by miRNAs (e.g., miR-10a/b, miR-143-3p, and miR-221-3p), which target the mRNA’s 3’UTR to suppress translation (3, 23, 24), EZH2-mediated epigenetic silencing through promoter methylation (25), and the transcriptional repressor ZNF667 (26). Post-translational regulation includes modulation of stability and activity through heterodimerization with SVBP, which protects VASH-1 from ubiquitin-mediated degradation, maintaining its catalytic function (4). Additionally, calcium-dependent calpain pathways influence its enzymatic activity (27). Alternative splicing generates isoforms with distinct functions: full-length VASH1A (365 aa) primarily regulates vascular normalization and chemosensitization (18), while truncated VASH1B (204 aa) enhances anti-angiogenic activity and induces autophagic cell death in endothelial cells (18, 28).

### 2.2 Core biological functions

VASH-1 performs a wide range of biological functions, with its central role being anti-angiogenesis. As an autocrine negative feedback regulator, it inhibits endothelial cell proliferation and migration induced by VEGF, FGF, and PDGF through paracrine signaling (1, 21). VASH-1 is notably enriched in the termination zones of nascent vessels, promoting vascular maturation and stabilization (7). Its expression level, particularly the VASH-1/CD31 ratio, serves as an indicator of neovascular density in tumors, such as neuroendocrine tumors, and correlates positively with the proliferation marker Ki-67 (29, 30). In addition to angiogenesis regulation, VASH-1 is involved in several other critical processes: Anti-fibrosis and organ protection, where it inhibits TGF-β-dependent renal fibroblast activation, reduces collagen deposition (8), preserves glomerular filtration barrier integrity (31), and suppresses endothelial-to-mesenchymal transition (EndMT) in retinal endothelial cells (32); Microtubule dynamics regulation, where as a tubulin carboxypeptidase (TCP), the VASH-1/SVBP complex catalyzes α-tubulin detyrosination, modulating cytoskeletal stability (5, 33), and indirectly blocking angiogenic signaling by inhibiting VEGFR2 endocytic trafficking (6); Inflammation and immune modulation, by downregulating the nuclear translocation of NF-κB pp65 and CCL2 expression, reducing monocyte/macrophage infiltration (9). In rheumatoid arthritis synovium, VASH-1 expression is dynamically regulated by inflammatory factors (34). Tumor microenvironment modulation is another significant function, where VASH-1 inhibits ovarian cancer cell migration (35) and reverses chemotherapy resistance in osteosarcoma by suppressing P-glycoprotein (P-gp) expression (36). VASH-1 exhibits tissue-specific and paradoxical functions, such as pro-angiogenic activity in diabetic penile tissue (10) and vascular demand-responsive expression in skeletal muscle (p36/42 isoforms). Under hypovascularized conditions, high expression of VASH-1 suppresses excessive angiogenesis, while post-exercise downregulation permits adaptive neovascularization (11).

### 2.3 Core molecular mechanisms of function

VASH-1’s diverse functions are driven by its enzymatic activity and critical regulation of key signaling pathways. At the core of its mechanism is its TCP activity: the VASH-1/SVBP heterodimer, dependent on the conserved Cys-His-Ser catalytic triad, specifically hydrolyzes the C-terminal tyrosine residue of α-tubulin (37). This enzymatic function directly: maintains podocyte microtubule integrity for renal protection (31) and disrupts VEGFR2 endocytosis and recycling to inhibit pro-angiogenic signaling (6). At the signaling pathway level, VASH-1 exerts broad effects *via*: Anti-fibrosis, by blocking Smad3 phosphorylation to inhibit TGF-β/Smad3 signaling and reduce collagen synthesis (8, 9); Anti-inflammation, by suppressing NF-κB pp65 nuclear translocation to downregulate pro-inflammatory genes (9); Chemoresistance reversal, through activation of AKT signaling to suppress P-gp expression (36); Cell death regulation, by inhibiting ferroptosis (*via* GPX4 upregulation) (38) or promoting protective mitophagy (*via* PINK1/Parkin pathway disruption) (39), with context-dependent effects. Tissue-specific microenvironments dynamically modulate VASH-1’s functional outputs, such as pro-angiogenic effects in diabetic penile tissue (10) and bidirectional vascular regulation by distinct isoforms (e.g., p36/p42) in muscle tissue (11).

**Figure 1:**
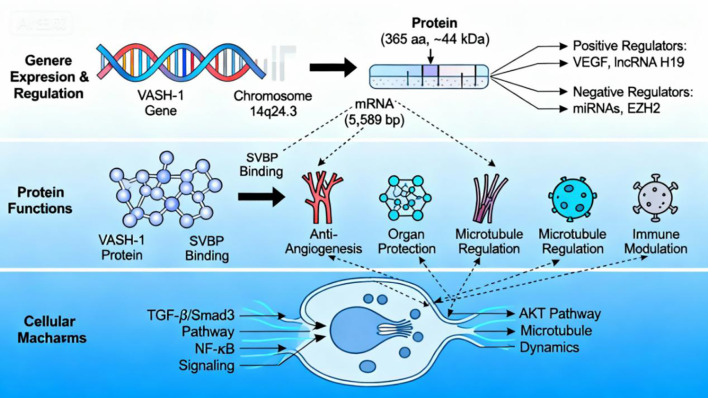
Molecular mechanism of Vasohibin-1. The figure conceptually outlines the molecular mechanism of VASH-1 in a hierarchical manner: Upper layer: the genetic origin and multi-level regulation of VASH-1 expression; Middle layer: the core biological functions of the VASH-1 protein, which depend on SVBP binding; Lower layer: key intracellular signaling pathways (TGF-β/Smad3, NF-κB, AKT) and molecular processes (microtubule dynamics) that mechanistically link VASH-1 activity to its observed functions.

In summary, VASH-1 synthesis, stability, and function are controlled by a tripartite regulatory network involving gene expression, protein interactions, and signaling pathways. Its pleiotropic biological functions—anti-angiogenesis, organ protection, microtubule remodeling, and inflammation modulation—are rooted in its core enzymatic activity and its deep integration with key signaling cascades, including TGF-β, NF-κB, and AKT. The dynamic remodeling of VASH-1’s functional phenotype by tissue-specific microenvironments not only highlights its biological complexity but also provides key theoretical frameworks and intervention opportunities for developing precision therapeutics targeting VASH-1 (**Figure 1**).

## 3 Expression profile and functional regulation of VASH-1 in diseases

### 3.1 Tumor diseases: from paradoxical expression to precision classification

#### 3.1.1 Neuroendocrine neoplasms

Neuroendocrine Neoplasms (NENs): VASH-1 is systematically downregulated in NENs, with serum levels (218.8 pg/mL) significantly lower than in healthy controls (973.1 pg/mL). Pancreatic NENs show higher expression compared to other subtypes (12). Within the tumor microenvironment, VASH-1 accumulates in neovascular endothelial cells, and the VASH-1/CD31 ratio increases with higher WHO malignancy grades (29). Notably, neuroendocrine regions exhibit significantly stronger VASH-1 expression than non-neuroendocrine areas (p < 0.0001), inversely correlating with serotonin levels (r = −0.19), suggesting VASH-1’s role in suppressing pathological angiogenesis and neuroendocrine activity (12, 40).

#### 3.1.2 Bladder cancer and gastric cancer

1. Bladder Cancer: VASH-1 is overexpressed in both tumor cells and vascular endothelium in bladder cancer, positively correlating with advanced stage (pT3-4), high pathological grade (G3), and distant metastasis (P < 0.01) (13). Paradoxically, high microvessel density (≥40/mm²) predicts chemotherapy sensitivity and improved survival (5-year recurrence-free survival: 66.3% vs 33.3%) (41). Its expression is dynamically regulated by the hypoxia-HIF-1α-VEGF axis: hypoxia downregulates VASH-1 in cancer cells, while tumor-associated macrophages (TAMs) reverse this suppression (42).
2. Gastric Cancer: In gastric cancer, VASH-1 positivity in tumor cells correlates with lymphovascular invasion (Ly^+^), advanced T-stage, and poor prognosis (43). TAMs upregulate VASH-1 expression *via* TGF-β/BMP signaling, thereby promoting angiogenesis and an immunosuppressive microenvironment (44, 45).

#### 3.1.3 Dual roles in breast and ovarian cancers

1. Breast Cancer: VASH-1 expression influences therapeutic responses: higher VASH-1 positivity rates (VPR) correlate with sensitivity to endocrine therapy (46), while low expression in triple-negative breast cancer (TNBC) predicts pathological complete response (pCR) to neoadjuvant chemotherapy (NAC) (47). miR-4530-mediated suppression of VASH-1 promotes angiogenesis (48).
2. Ovarian Cancer: VASH-1 exhibits paradoxical roles: Tumor-suppressive: Low tissue expression correlates with poor prognosis, and its paracrine secretion inhibits endothelial migration and proliferation (2, 49). Pro-tumorigenic: In serous carcinoma, VASH-1 strongly correlates with the metastasis gene MACC1 (r = 0.518), serving as an independent poor prognostic factor (RR = 2.185) (50). Targeting VASH-2 enhances paclitaxel sensitivity (51).

#### 3.1.4 Spatial paradox in hepatocellular and colorectal cancers

(1) HCC: In HCC, VASH-1 positivity is higher in tumor tissue (38.5%) compared to adjacent tissue (16.2%), correlating with microvascular invasion (P = 0.02) and shorter survival (HR = 2.554) (52, 53). Cancer-associated fibroblasts (CAFs) epigenetically silence VASH-1 *via* the VEGF-EZH2 axis to promote angiogenesis (54).

(2) CRC: VASH-1’s role in CRC depends on its spatial localization: Pro-metastatic: High cytoplasmic expression in cancer cells promotes advanced metastasis (14, 55). Anti-metastatic: High stromal expression suppresses metastasis (15). The circASS1/miR-1269a axis targets VASH-1 for suppression (56).

Summary: VASH-1 exhibits microenvironment-dependent functionality in tumors: Protumorigenic overexpression occurs in HCC and bladder cancer, driven by the hypoxia-HIF-1α axis, while anti-tumorigenic expression is observed in ovarian cancer through paracrine endothelial suppression and in CRC stroma. Spatially, cancer cells typically promote progression, whereas the stroma may inhibit metastasis. As a circulating biomarker, low serum levels assist in NEN diagnosis, while elevated plasma levels are associated with reduced mortality risk in non-small cell lung cancer (NSCLC) (12, 57) (**Table 1**).

**Table 1:** Common patterns in other tumors.

Tumor type	Core finding	Key mechanism	Clinical trial	Refs
Osteosarcoma	Low tumor expression → doxorubicin resistance	AKT-P-gp pathway inhibition	Not specified	(36)
NSCLC	High tissue expression → poor prognosis; High plasma levels → ↓mortality	miR-143-3p targeting	Yes; n = 79	(57)
Esophageal cancer	High cancer cell expression → ↓tumor volume; High plasma levels → ↑lymph node metastasis	Direct proliferation/migration inhibition	Yes; n = 100	(58)
Prostate cancer	Ductal adenocarcinoma > acinar adenocarcinoma expression → Gleason upgrade, ↓survival	Undefined	Yes; n = 34	(59)
HNSCC	High tumor/endothelial expression → ↑lymph node recurrence, ↓survival	Undefined	Yes; n = 61	(60)
LAM	Correlates with VEGFR2/MMP9/p-mTOR	Pro-angiogenic	Yes; n = 36	(61)

The symbol → indicates that A causes B, ↓ indicates downregulation (decreased expression), and ↑ indicates upregulation (increased expression).

### 3.2 Immunological disorders: guardian of vascular homeostasis

#### 3.2.1 Systemic sclerosis

SSc is a multisystem autoimmune disease characterized by widespread small-vessel vasculopathy and damage, accompanied by activation of skin and organ fibrosis (62). It involves a complex interplay of vasculopathy, inflammation, and fibrosis (63). In SSc, serum VASH-1 levels are significantly elevated (P < 0.01) and positively correlate with modified Rodnan skin thickness scores (mRSS; r = 0.48), with higher concentrations observed in diffuse cutaneous SSc (dcSSc) and patients with interstitial lung disease (8). Mechanistically, VASH-1 inhibits fibroblast activation by suppressing the TGF-β/Smad3 pathway, reducing collagen I/III deposition, and establishing an anti-fibrotic feedback loop.

#### 3.2.2 Atopic dermatitis

AD is an inflammatory skin disease affecting up to 20% of children and 5% of adults (64). Genetic factors involved include those related to skin-barrier formation (e.g., filaggrin gene mutations), epidermal enzyme activity, lipid metabolism, and immune-system regulation (65). Patients with AD exhibit significantly higher serum VASH-1 levels than healthy controls (P < 0.001), with specific endothelial overexpression in lesional skin (66). VASH-1 expression correlates positively with disease duration and lesional VEGF-A levels (r = 0.72), suggesting a role in negative feedback regulation of VEGF-driven angiogenesis to maintain chronic inflammatory homeostasis. This mechanism requires further validation.

#### 3.2.3 Rheumatoid arthritis

RA is a heterogeneous, prevalent, and chronic autoimmune disease characterized by joint pain, swelling, and significant disability (67). In RA, inflammatory cytokines play a pivotal role in triggering abnormal osteoclastogenesis, leading to joint destruction (68). RA is associated with immune-system dysregulation and persistent inflammation (69).

VASH-1 is highly expressed in synovial lining fibroblasts and pannus endothelium of patients with RA (34). Expression during active disease exceeds that during remission and strongly correlates with synovitis scores (r = 0.842). Induced by VEGF, TNF-α, and IL-6, VASH-1 forms a negative feedback loop to constrain pathological pannus expansion.

### 3.3 Other diseases: trans-organ protection and dysregulation

#### 3.3.1 Renal diseases

(1) DKD is the most common microvascular complication of both type 1 and type 2 diabetes, now surpassing glomerulonephritis as the leading cause of chronic kidney disease (CKD) and end-stage renal disease (ESRD) (70). Its pathogenesis involves synergistic metabolic, hemodynamic, inflammatory, and fibrotic pathways. Renal tissue VASH-1 expression is reduced and correlates positively with renal impairment, while serum levels increase with worsening proteinuria (71–73). Mechanistically, intrarenal VASH-1 deficiency reduces podocyte α-tubulin detyrosination, disrupting the filtration barrier (31). (2) Renal fibrosis is a common terminal pathological manifestation in the progression of CKD (74) and is a key independent risk factor for CKD progression and poor prognosis (75). In fibrosis, VASH-1 suppression by miR-10a/b targeting promotes Smad3 phosphorylation, exacerbating collagen deposition (3). (3) CKD: Renal VASH-1 is upregulated and localizes to glomerular endothelia, mesangial cells, and interstitial infiltrating polymorphonuclear neutrophils (PMNs). Its density correlates with crescent formation (r = 0.64) and interstitial inflammation scores. Elevated plasma and urinary VASH-1 levels predict rapid estimated glomerular filtration rate (eGFR) decline (76, 77).

#### 3.3.2 Metabolic and vascular diseases

(1) Diabetic Retinopathy (DR): DR, a common complication of diabetes and a leading cause of vision loss globally, can be prevented with early detection and intervention (78, 79). Serum VASH-1 concentrations follow a gradient: healthy controls < non-proliferative DR < proliferative DR (P < 0.05), positively correlating with body mass index (BMI) and glycated hemoglobin (HbA1c) (80). The flavonoid kaempferol protects retinal ganglion cells through the ERK/VASH-1 axis (81). (2) Cardiovascular Diseases: In heart failure, myocardial VASH-1 expression predominates (exceeding VASH-2 by more than 10-fold), mediating mitochondrial respiratory dysfunction (39, 82). Atherosclerotic plaque neovessels show high VASH-1 expression, which correlates with VEGFA (r = 0.788) and VCAM1 (r = 0.94) (83). (3) Pulmonary Hypertension (PAH) & Idiopathic Pulmonary Fibrosis (IPF): In PAH, elevated VASH-1 facilitates vascular remodeling, with an imbalanced VASH-2/VASH-1 ratio contributing to disease progression (84). In IPF, increased VASH-1 suppresses EndMT (85).

#### 3.3.3 Pregnancy and reproductive disorders

(1) Preeclampsia (PE): A common pregnancy-related complication, PE is a leading cause of maternal, fetal, and neonatal morbidity and mortality, with long-term adverse effects on both mother and offspring (86). First-trimester serum VASH-1 is significantly elevated (cutoff: 1314.73 pg/mL, AUC = 0.631) (16). Placental VASH-1 expression is induced by hypoxia-HIF-1α (87). (2) Placental Development: Endothelial VASH-1 inhibits fetal angiogenesis, while trophoblastic VASH-2 promotes syncytiotrophoblast fusion (88). (3) Erectile Dysfunction (ED): Affecting more than 50% of men over 40 years of age (89), ED, defined as the inability to achieve or maintain an erection firm enough for satisfactory sexual performance, is linked to risk factors such as hypertension, hypercholesterolemia, obesity, sedentary lifestyle, diabetes, and smoking (90). Diabetic cavernosal VASH-1 downregulation contributes to microvascular dysregulation (10). (4) Endometriosis: miR-143-3p-mediated suppression of VASH-1 promotes stromal cell invasion (91).

#### 3.3.4 Aging and injury repair

**Figure 2:**
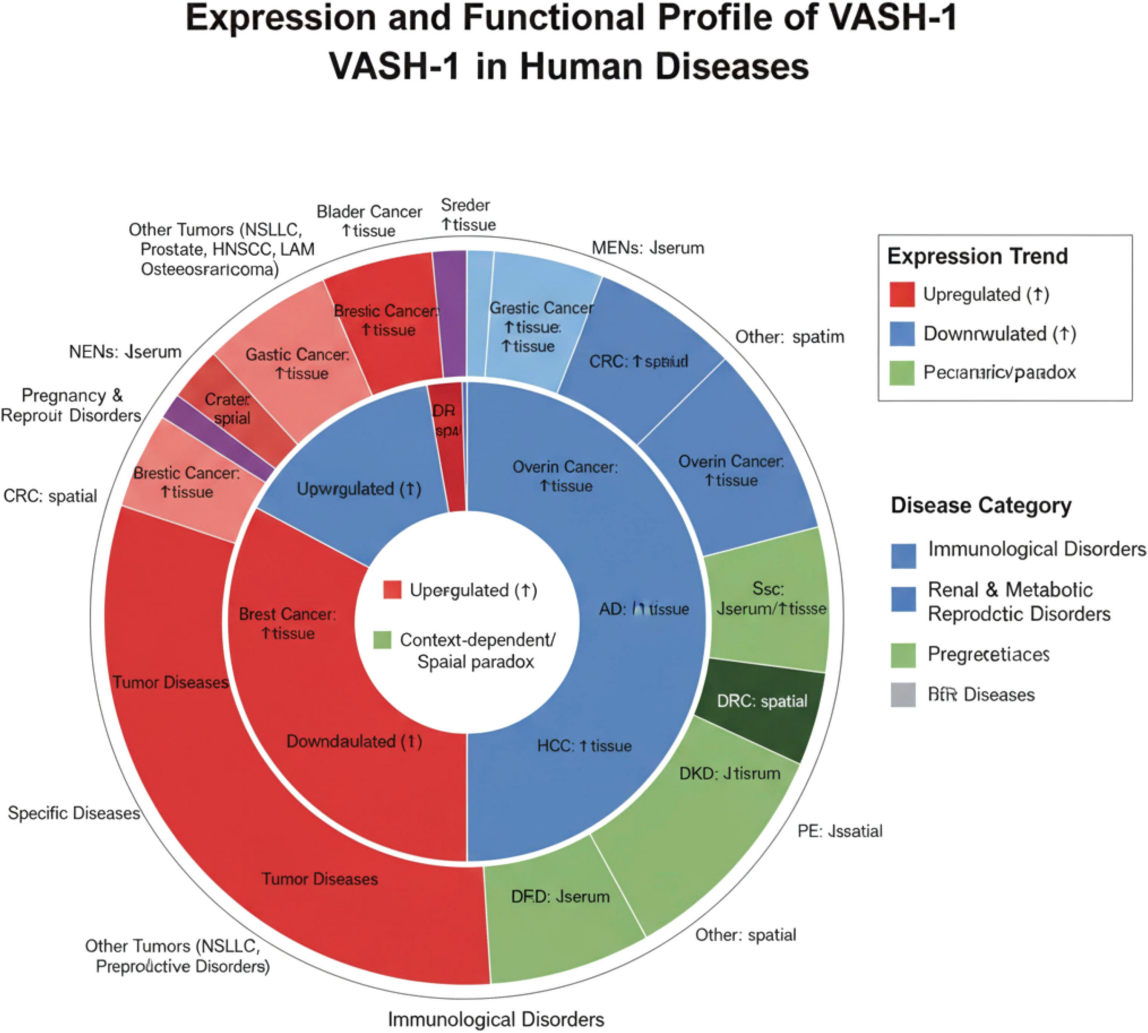
Schematic diagram of VASH-1 expression in various diseases. Horizontal bars indicate expression direction (↑ up-regulation, ↓ down-regulation) in the indicated disease entities; bar color denotes specimen source (tissue, serum, or spatial distribution). Data integrate oncologic (gastric, colorectal, HCC, prostate, etc.), metabolic-vascular (DKD, DR, CVD), obstetric (preeclampsia), fibro-inflammatory (SSc, IPF), reproductive, aging, and injury-repair contexts, highlighting the context-dependent and trans-organ protective/dysregulatory nature of VASH-1.

(1) Vascular Aging: Senescent endothelia show VASH-1 downregulation *via* miR-22-3p targeting (92). VASH1(−/−) mice exhibit increased lifespan, associated with reduced insulin resistance (93). (2) Age-Related Macular Degeneration (AMD): AMD, a degenerative disease causing central vision loss, is classified into dry (non-neovascular) and wet (neovascular) types (94). Hypoxia-induced downregulation of retinal pigment epithelium (RPE) VASH-1 promotes choroidal neovascularization through increased tRF-Glu-CTC expression (95). (3) Cerebral Ischemia-Reperfusion Injury: Vash1(+/-) mice show reduced infarct volume *via* upregulation of GPX4 (inhibiting ferroptosis) and downregulation of ACSL4 (reducing pro-ferroptotic signaling) (38). (4) Bronchiolitis Obliterans (BO): BO, an irreversible obstructive lung disease characterized by terminal bronchiole inflammation and fibrosis (96), follows a pathogenesis cascade of epithelial injury → aberrant inflammation → fibroproliferation → luminal obliteration (97). VASH-1 inhibits graft-associated pathological angiogenesis, attenuating tracheal fibrosis (21) **Figure 2**.

## 4 Clinical translational value of vasohibin-1 (VASH-1): from diagnostic biomarker to precision intervention

### 4.1 Tumor diseases: navigator for diagnostic stratification and therapeutic response

#### 4.1.1 Prognostic alert of tissue biomarkers

(1) In bladder cancer, a highly recurrent urological malignancy, immunohistochemistry was performed on 50 tumor specimens to assess the expression of VASH-1 and angiogenesis-related factors VEGF, HIF-1α, and CD34, correlating their levels with clinicopathological features. Elevated VASH-1 expression emerged as an indicator of aggressive phenotype, significantly associated with advanced stage (pT3-4), high grade (G3), and metastatic risk (P < 0.01), functioning as an independent marker of poor prognosis (13). (2) In gastric cancer, a study analyzed VASH-1 immunohistochemical expression in 210 patients who underwent radical gastrectomy, classifying them into VASH-1–positive and VASH-1–negative groups. VASH-1 positivity in cancer cells was strongly correlated with lymphovascular invasion (Ly^+^), T-stage progression, and reduced overall survival (43). (3) In HCC, a study evaluated the immunoreactivity of FGF-2 and VEGF-A, along with microvessel density (MVD-CD34) as defined by VASH-1 and CD34 expression in 181 patients with HCC, correlating these findings with clinical outcomes. Double immunostaining for CD34, VASH-1, and Ki-67 was conducted to assess the angiogenic activity of endothelial cells. A VASH-1/CD34 ratio >0.459 predicted microvascular invasion (53). (4) In ovarian serous carcinoma, immunohistochemical Elivision™ staining was used to detect VASH-1, MACC1, and KAI1 proteins in 124 serous ovarian cancer tissues and 30 serous cystadenoma controls. VASH-1 strongly correlated with the metastasis-associated gene MACC1 (r = 0.518) and served as an independent poor-prognostic factor (relative risk [RR] = 2.185) (50).

#### 4.1.2 Predictors of therapeutic response

(1) In a paradoxical study involving 40 Japanese patients with BCa who received NAC followed by radical cystectomy, immunohistochemical expression of CD34, VASH-1, and carbonic anhydrase 9 (CA9) was compared between tumors that achieved complete pathological clearance (ypT0) and those with residual disease. Within the bladder cancer microvasculature, high VASH-1 expression (≥ 40/mm²) predicted chemosensitivity, indicating achievement of ypT0 after NAC and correlating with a higher 5-year recurrence-free survival rate (66.3% vs 33.3%) (41). (2) In TNBC, a study performed dual immunohistochemistry on biopsy specimens to quantify CD8^+^ and FOXP3^+^ tumor-infiltrating lymphocytes, along with immunolabeling for VASH-1, CD31, EGFR, CK5/6, and Ki-67. Low tumor VASH-1 expression predicted a positive response to chemotherapy, achieving 78% sensitivity and 82% specificity for pCR, with the pCR rate in the low-expression cohort being 3.5-fold higher (47).

#### 4.1.3 Breakthrough value of circulating biomarkers

A total of 79 patients with lung cancer (51 men, 28 women; age range 34–83 years; 46 adenocarcinomas, 27 squamous cell carcinomas, and 6 other histologies) who underwent pulmonary resection were enrolled. Preoperative plasma VASH-1 concentrations were measured and correlated with clinical characteristics and prognosis. Patients with NSCLC whose preoperative plasma VASH-1 exceeded 1190.4 fmol/mL had a 58% reduction in death risk compared to those with lower levels, highlighting its potential as a non-invasive biomarker (57) **Table 2**.

**Table 2:** Core diagnostic value of VASH-1 in tumors.

Tumor type	Sample type	Clinical significance	Clinical trial	Refs
Osteosarcoma	Tumor tissue	Low expression → ↑40% doxorubicin resistance	Not specified	(36)
Prostate ductal adenocarcinoma	Tumor tissue	High expression (density >45.1/mm²) → ↓30% 5-year survival	Yes; n = 34	(59)
Cervical squamous cell carcinoma	Lymphatic endothelium	→ ↑Lymph node metastasis risk (miR-221-3p targeting)	Yes; n = 107	(24)
Esophageal squamous cell carcinoma	Tumor tissue	High expression → ↓5-year survival	Yes; n = 209	(98)
HCC subtyping	Tumor tissue	Low expression → K19+ high-aggressiveness subtype → ↑metastasis risk	Yes; n = 136	(99)

### 4.2 Non-tumor diseases: sentinel for early warning and disease monitoring

#### 4.2.1 Serum-dominated early-warning systems

(1) In PE, a prospective case-control study collected first-trimester blood from 1,054 pregnant women, including 43 who later developed PE and 129 controls randomly selected (1:3) from 777 uncomplicated pregnancies. Commercial ELISA kits quantified serum VASH-1, cardiotropin-1, and endocan. VASH-1 levels were significantly higher in the PE group (p = 0.010), with ROC analysis yielding an AUC of 0.631 (95% CI 0.53–0.72) and an optimal cut-off of 1,314.73 pg/mL (60% sensitivity, 60% specificity), providing an early window for high-risk intervention (16). (2) In DKD, 143 patients with type-2 DN were compared to 80 diabetes-without-nephropathy controls. Stratifying DN by UACR (micro-albuminuria 30–300 mg/g; macro-albuminuria ≥ 300 mg/g) showed that serum VASH-1 levels rose in parallel with increasing UACR, validating it as a dynamic biomarker of renal deterioration (p < 0.01) (72). (3) In SSc, clinical correlation of serum VASH-1 with its dermal expression revealed a significant association with the mRSS (r = 0.48), indicating its predictive value for diffuse cutaneous SSc and interstitial lung disease (8).

#### 4.2.2 Tissue-specific injury markers

**Figure 3:**
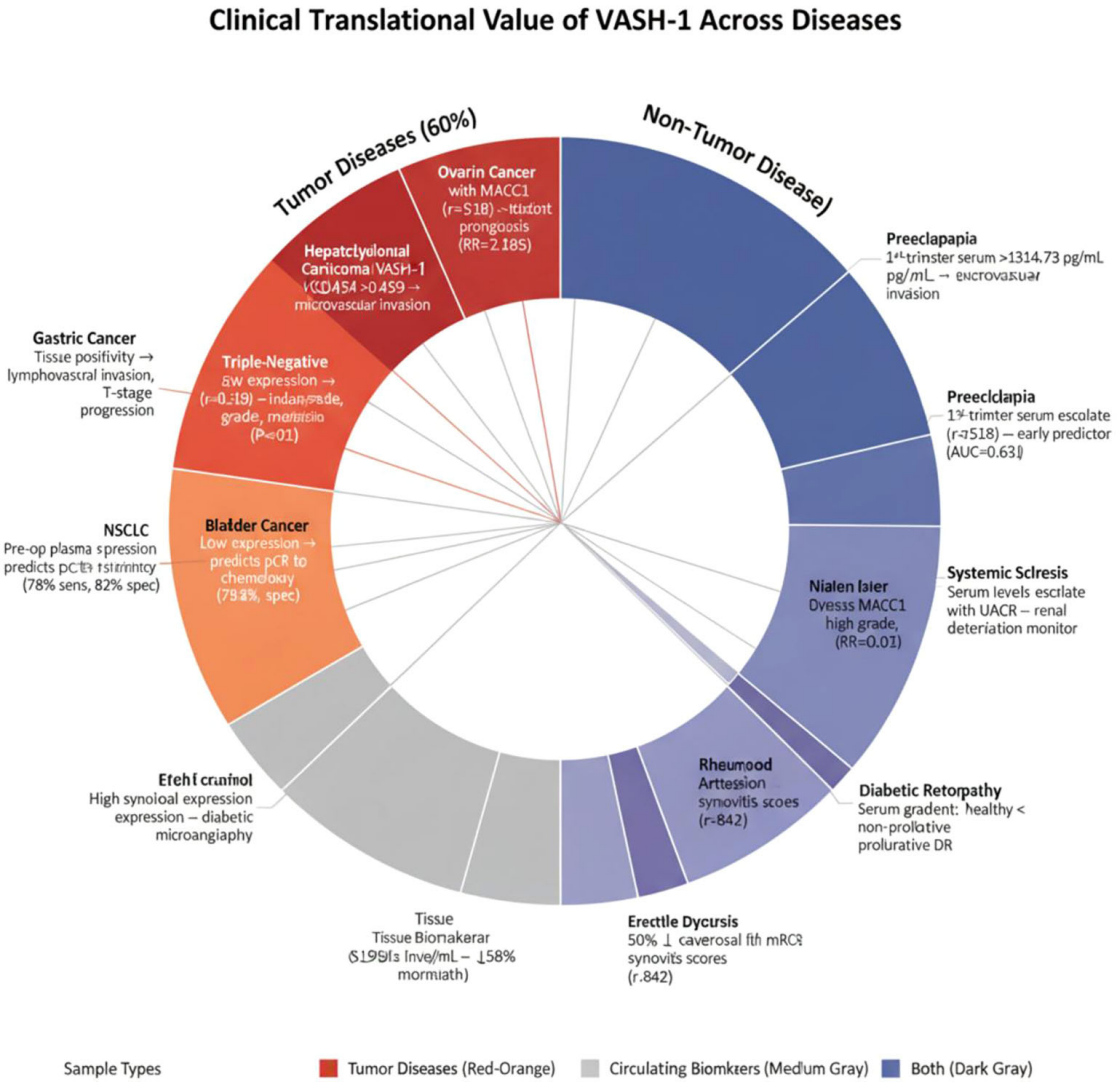
Diagnostic value of VASH-1 in multiple diseases. Colored bars summarize its diagnostic or risk-stratifying value in malignancies (ovarian, gastric, NSCLC, bladder, HCC), obstetric (first-trimester preeclampsia), metabolic-microvascular (DKD, DR), fibro-inflammatory (SSc, RA) and reproductive (ED) disorders. Arrow direction (↑ or ↓) indicates whether high or low expression predicts poor outcome; adjacent numbers give AUC, sensitivity/specificity, relative risk or correlation coefficient. Sample source (tissue, serum, synovial fluid, cavernosal biopsy) is coded by bar shade, illustrating VASH-1’s utility as both a circulating early-warning sentinel and a tissue-specific injury marker.

(1) In ED, a study found that VASH-1, traditionally viewed as anti-angiogenic, significantly restored erectile function in diabetic mice. Compared to healthy controls, diabetic patients exhibited a 50% reduction in corporal VASH-1 expression, marking a threshold indicative of diabetic microangiopathy (10). (2) In RA, surgical synovial samples from patients with RA and osteoarthritis (OA) were examined by immunohistochemistry for VASH-1 distribution and correlated with synovial inflammation. *In vitro*, RA synovial fibroblasts (RASFs) stimulated with VEGF or pro-inflammatory cytokines under normoxia or hypoxia were analyzed by real-time PCR for VASH-1 and VEGF mRNA. High synovial VASH-1 expression strongly correlated with synovitis score (r = 0.842), positioning it as an activity biomarker of synovial inflammation (34) **Table 3**; **Figure 3**.

**Table 3:** Extended diagnostic value in Non-tumor diseases.

Disease type	Sample type	Clinical significance	Key mechanism	Clinical trial	Refs
Adolescent hypertension	Circulating blood	High expression → systolic BP correlation (r = 0.314)	Early vascular injury marker	Yes; n = 132	(100)
Coronary artery disease (CAD)	Circulating EPCs	Low expression → vascular repair dysfunction (miR-720 targeting)	Endothelial repair warning	Not specified	(101)
Periodontitis	Gingival tissue	High expression → Pg-induced inflammatory response	Pathogen response marker	Not specified	(102)
Hypertension target organ injury	Circulating blood	↑Predictive power combined with miR-335-5p/HYAL1	Multi-marker synergy	Yes; n = 132	(100)
Diabetic retinopathy	Serum ↑	Staging marker (proliferative > non-proliferative)	↑Levels in the proliferative phase	Yes; n = 162	(80)

### 4.3 Therapeutic targets: from mechanism to clinical intervention

#### 4.3.1 Renal diseases: anti-fibrotic breakthroughs

**Figure 4:**
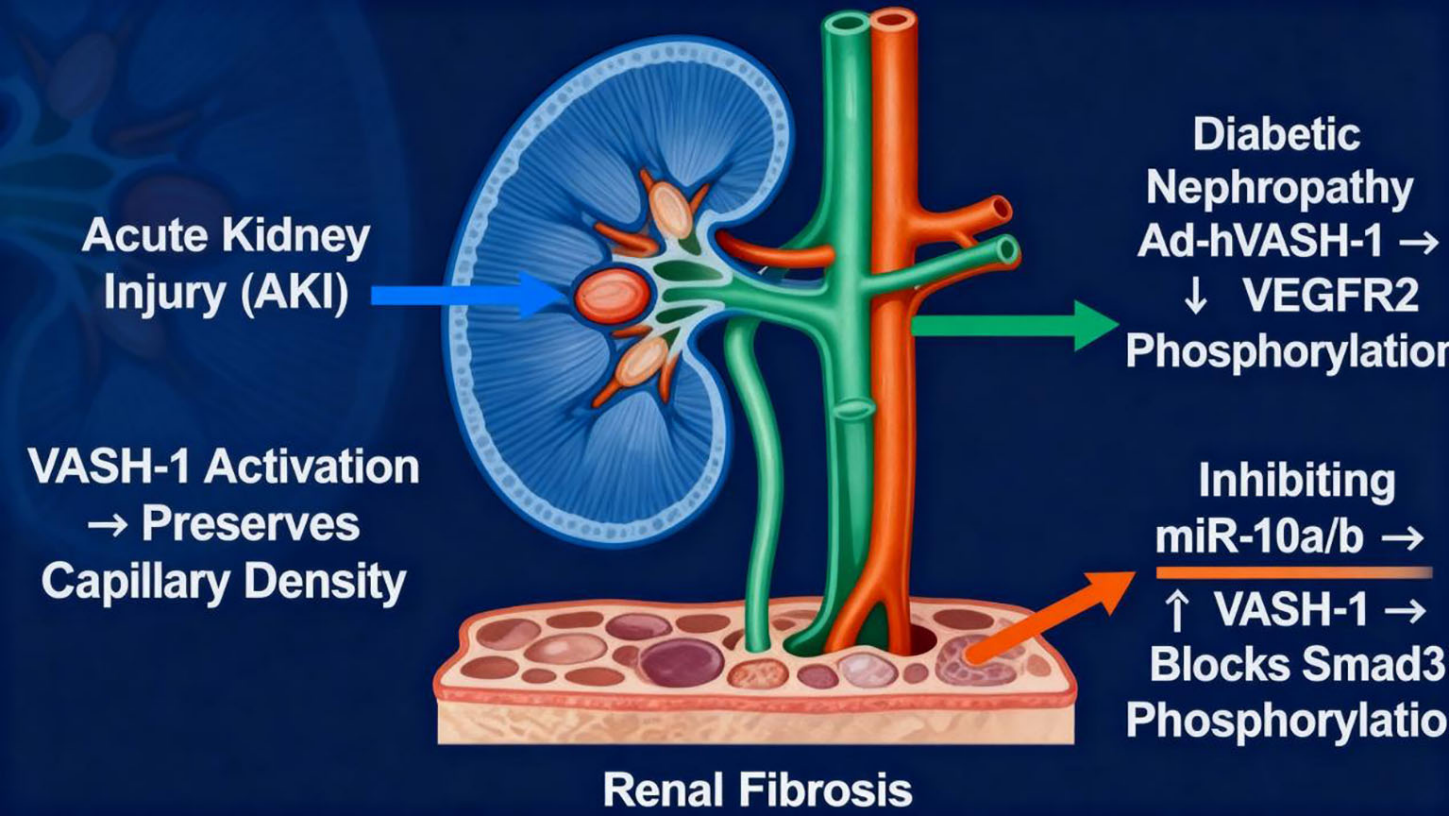
VASH-1-mediated therapeutic mechanisms in renal diseases. The schematic shows three injury models: acute kidney injury (AKI), diabetic nephropathy (DN) and renal fibrosis. In AKI, activated VASH-1 maintains peritubular capillary density and lowers VEGFR2 phosphorylation, reducing inflammatory infiltration and tubular damage. In DN, Ad-hVASH-1 gene delivery suppresses VEGFR2 signaling and down-regulates TGF-β1/MCP-1, producing a 70% decrease in proteinuria. In fibrosis, blockade of miR-10a/b restores VASH-1 levels, prevents Smad3 phosphorylation and cuts collagen deposition by ~45%. Collectively, the figure illustrates VASH-1 as a multi-hit therapeutic target that simultaneously preserves microvasculature, antagonizes TGF-β/Smad3 signaling and attenuates extracellular-matrix accumulation in diverse renal pathologies.

(1) Acute Kidney Injury (AKI): VASH-1 activation in cisplatin-induced AKI preserves capillary density, resulting in a 40% reduction in inflammatory infiltration and a 60% reduction in tubular injury score (103). (2) DN: Adenovirus-delivered human VASH-1 (Ad-hVASH-1), administered intravenously, reduces VEGFR2 phosphorylation and TGF-β1/MCP-1 expression, leading to a 70% improvement in proteinuria (17). (3) Renal Fibrosis: Inhibition of miR-10a/b increases VASH-1 expression, preventing Smad3 phosphorylation and reducing collagen deposition by approximately 45% (3). These findings highlight the potential of VASH-1 as a therapeutic target in various renal disease models **Figure 4**.

#### 4.3.2 Tumor therapy: reversing resistance and vascular remodeling

**Figure 5:**
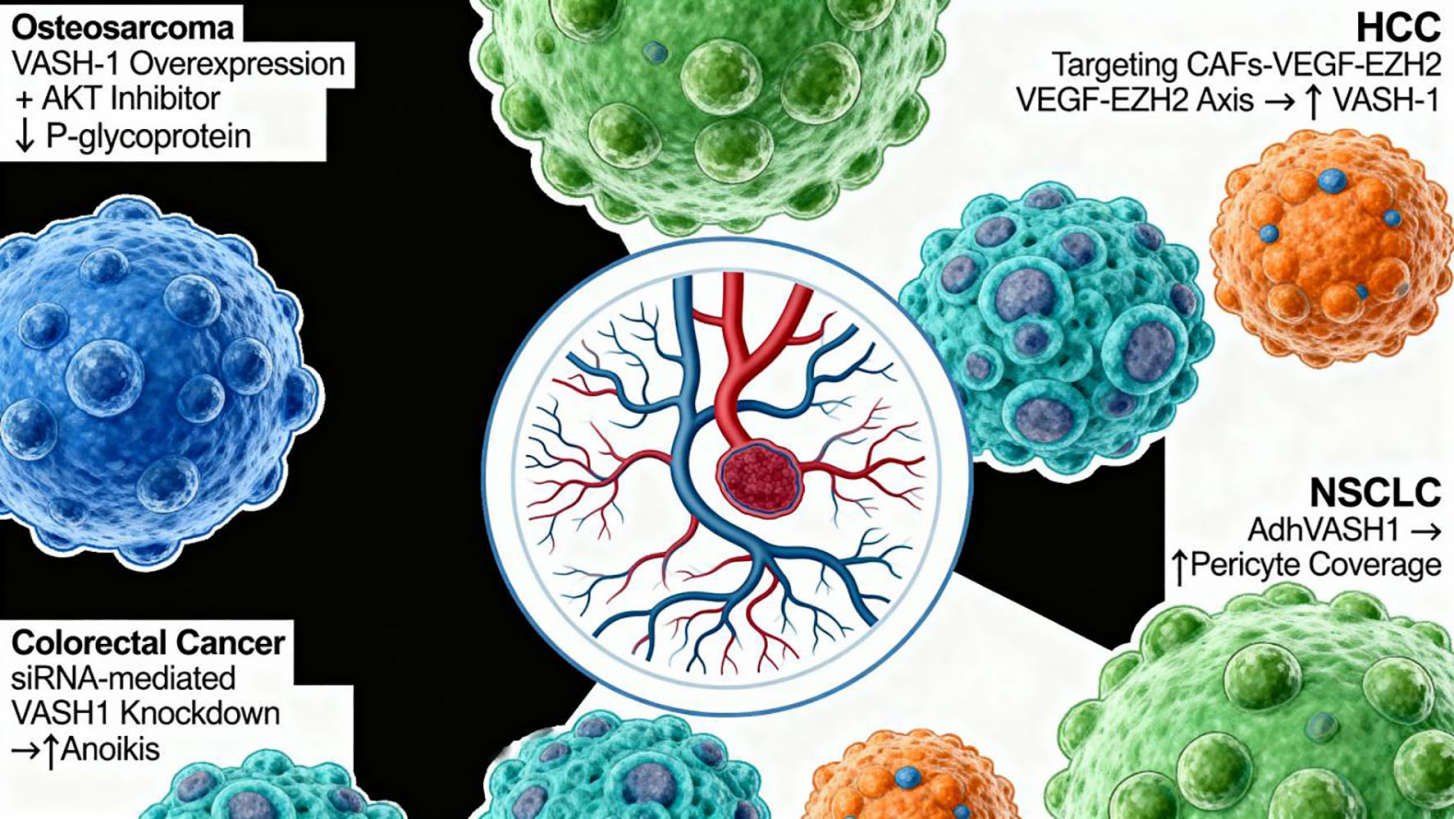
Multi-faceted therapeutic targeting of VASH-1 in cancer treatment. The panel illustrates five tumor models: osteosarcoma, HCC, CRC, NSCLC and ovarian cancer. Osteosarcoma: VASH-1 overexpression plus AKT inhibitor down-regulates P-glycoprotein, halves doxorubicin IC_50_; and re-sensitizes cells. HCC: Disruption of the CAF-VEGF-EZH2 axis boosts endogenous VASH-1, curtails neovascularization and shrinks primary tumors by ~65%. CRC: siRNA knock-down of VASH-1 triggers anoikis and reduces liver metastases by ~70%. NSCLC: Ad-hVASH-1 increases pericyte coverage, normalizes vessels and, combined with cisplatin, suppresses xenograft growth by ~80%. Ovarian cancer: CRISPR deletion of VASH-2 (functional counterpart) decreases microtubule detyrosination, elevates cyclin B1 and lowers paclitaxel IC_50_; by ~40%. Collectively, the figure portrays VASH-1 as a druggable node that can be either up-regulated or down-regulated to reverse chemo-resistance, re-engineer the tumor vasculature and improve therapeutic indices across divergent malignancies.

(1) Osteosarcoma: VASH-1 overexpression combined with AKT inhibitor treatment reduces P-gp expression, enhancing doxorubicin accumulation and reversing resistance (IC50 reduced by approximately 50%) (36). (2) HCC: Targeting the CAFs-VEGF-EZH2 axis increases VASH-1 expression, inhibiting angiogenesis and reducing primary tumor volume by approximately 65% (54). (3) CRC: siRNA-mediated VASH-1 knockdown promotes anoikis, reducing liver metastases by approximately 70% (55). (4) NSCLC: Intravenous administration of adenovirus-delivered human VASH-1 (Ad-hVASH-1) enhances pericyte coverage, promoting vascular maturation. Combined with cisplatin, it suppresses tumor growth by approximately 80% in nude mice (104). (5) Ovarian Cancer: CRISPR-mediated VASH-2 knockout reduces microtubule detyrosination and increases cyclin B1 expression, enhancing paclitaxel sensitivity (IC50 reduced by approximately 40%) (51) **Figure 5**.

#### 4.3.3 Cardio-cerebrovascular protection

**Figure 6:**
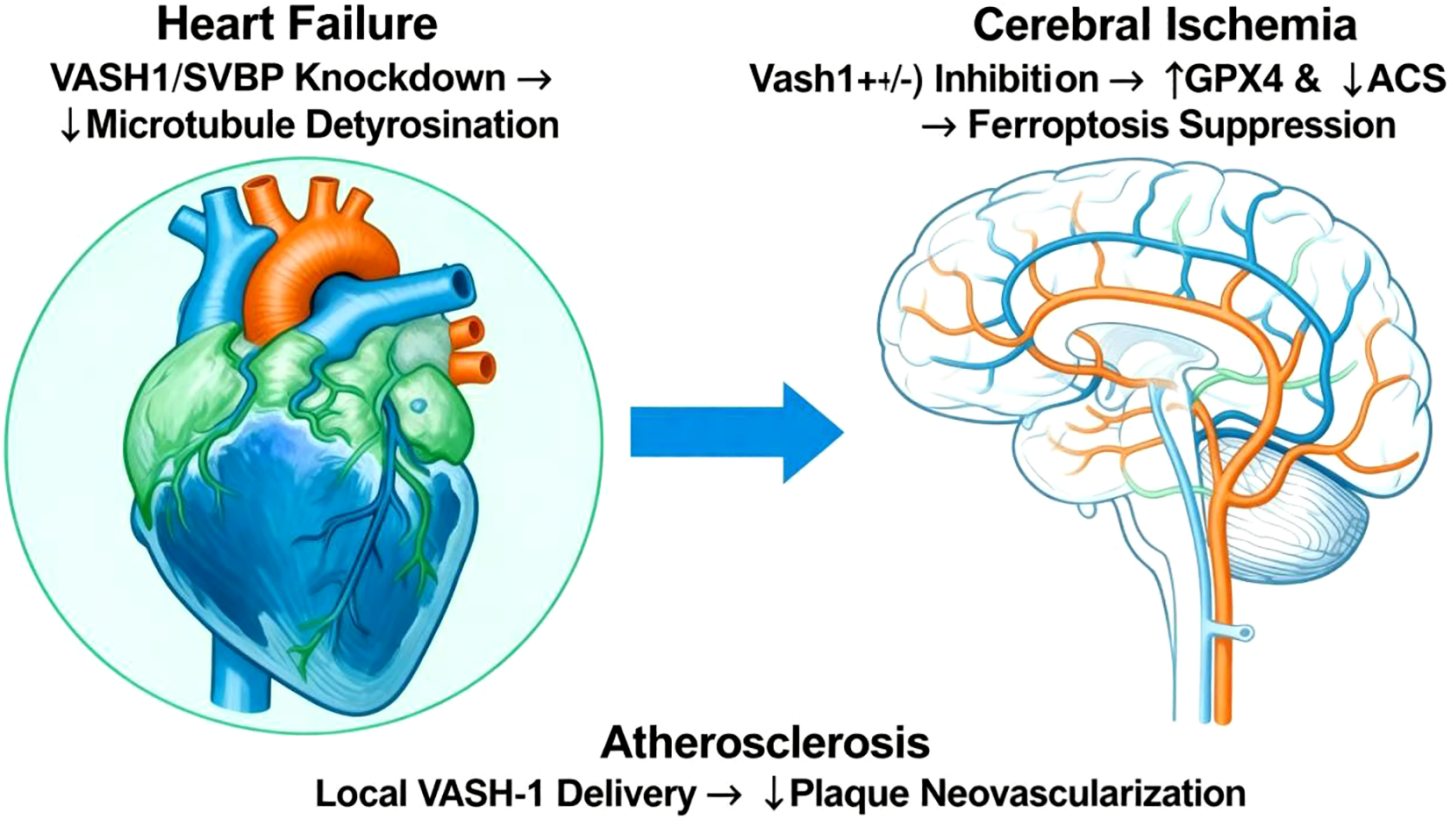
Cardio-cerebrovascular protective effects of VASH-1 modulation. The panel depicts three injury scenarios: cerebral ischemia, heart failure and atherosclerosis. Cerebral ischemia: partial Vash1 knock-down up-regulates GPX4, down-regulates ACSL4, suppresses microtubule de-tyrosination-driven ferroptosis and cuts infarct volume by ~30%. Heart failure: VASH-1/SVBP silencing decreases microtubule de-tyrosination, reduces myocardial stiffness and improves diastolic function by ~35%. Atherosclerosis: local VASH-1 delivery attenuates plaque neovascularization and lowers rupture risk by ~50%. Together, the figure positions VASH-1 as a druggable axis that simultaneously limits ferroptotic death, improves myocardial compliance and stabilizes atheromatous plaques, offering multi-level cardio-cerebrovascular protection.

(1) Cerebral Ischemia: Vash1(+/-) inhibition upregulates GPX4 and downregulates ACSL4, inhibiting ferroptosis and reducing infarct volume by approximately 30% (38). (2) Heart Failure: VASH-1/SVBP knockdown reduces microtubule detyrosination, decreasing myocardial stiffness and improving diastolic function by approximately 35% (82). (3) Atherosclerosis: Local VASH-1 delivery reduces plaque neovascularization, lowering rupture risk by approximately 50% (83). These findings demonstrate the therapeutic potential of targeting VASH-1 in oncology and cardiovascular disease contexts **Table 4**; **Figure 6**.

**Table 4:** VASH-1 intervention in fibrotic diseases.

Disease	Intervention	Core mechanism	Efficacy	Model	Refs
Pulmonary fibrosis	Gambogic acid	↑VASH-1/VASH-2 ratio → ↓TGF-β1/Smad3	↓60% collagen deposition	Bleomycin mice	(85)
Portal hypertension	Adenovirus-mediated VASH-1 OE	Breaks the VEGF/VASH-1 negative feedback loop	↓40% portal pressure	Cirrhotic rats	(105)
Corneal neovascularization	Subconjunctival Ad-VASH-1	↓Vegfr2 expression → angiogenesis inhibition	↓50% neovascular area	Alkali-burn rats	(106)

### 4.3.4 Cutting-edge technology platforms: precision delivery and intelligent modulation

### 4.4 Technological highlights

**Figure 7:**
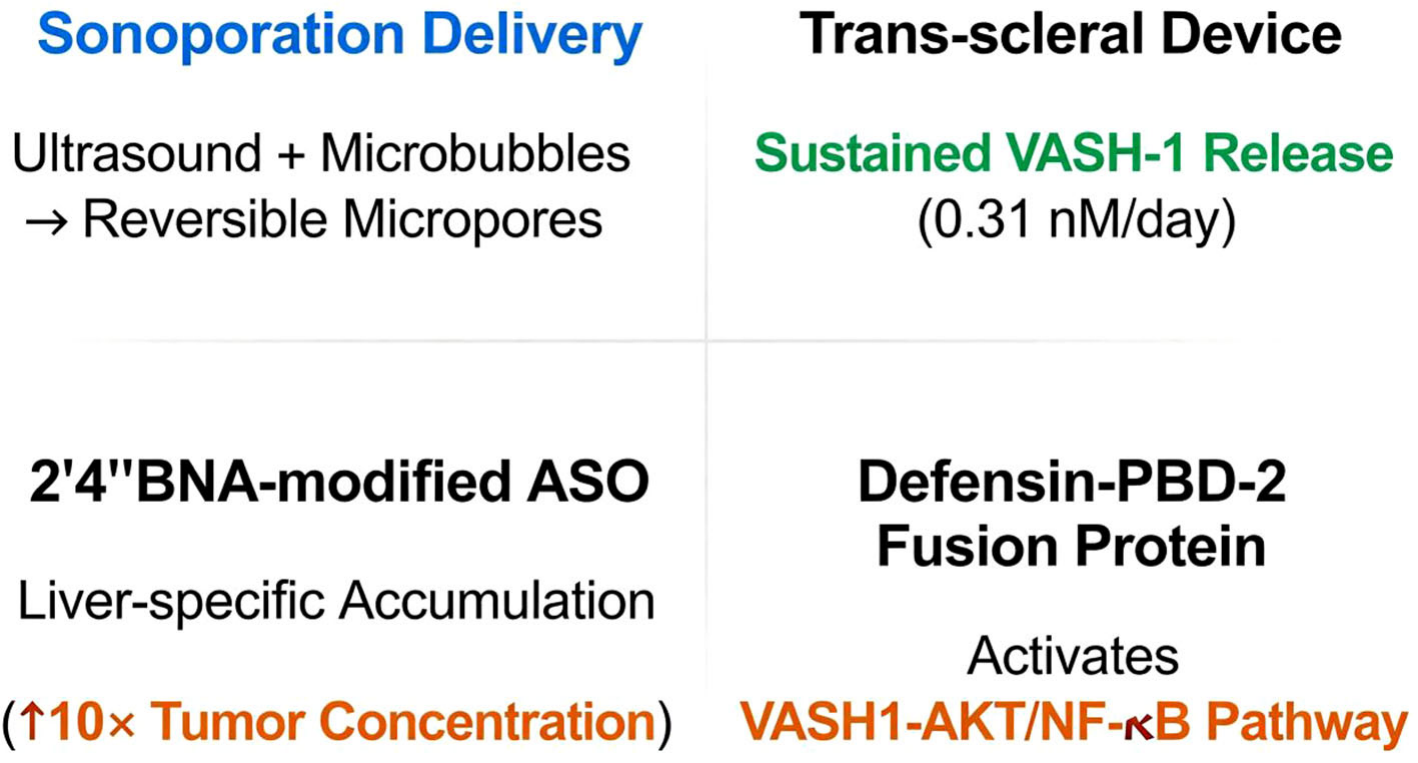
Cutting-edge technological platforms for VASH-1 targeted therapy. The panel illustrates four technologies: Sonoporation: ultrasound + microbubbles create reversible endothelial micropores, enabling alternating transfection of VASH1A (pro-normalization) and VASH1B (pro-pruning) for spatiotemporal vascular control. Trans-scleral device: reservoir releases VASH-1 at 0.31 nM/day, maintaining therapeutic levels >28 days against choroidal neovascularization. 2’,4’-BNA-modified ASO: locked nucleic acid chemistry targets VASH2 mRNA, producing 10-fold higher drug accumulation in HCC with minimal off-target exposure. Defensin-PBD-2 fusion protein: activates the VASH1-AKT/NF-κB pathway at inflammatory loci, boosting mouse sepsis survival by 60%. Collectively, the figure highlights how physical, chemical and biological engineering converge to achieve precise, sustained and intelligent modulation of the VASH-1 axis.

(1) Sonoporation, a physical delivery method utilizing ultrasound and microbubbles to create reversible micropores in cell membranes for efficient macromolecule delivery (110), enables bidirectional modulation of angiogenesis: VASH1A promotes vascular normalization to enhance drug delivery, while VASH1B effectively prunes aberrant vessels (18). (2) 2’,4’-BNA-modified antisense oligonucleotides (ASO), a third-generation ASO class with a bridged nucleic acid structure that locks the ribose in a 3’-endo conformation, offer high RNA affinity, nuclease resistance, and *in vivo* stability, minimizing off-target risks (111, 112). In HCC, this technology achieves liver-targeted accumulation, with a 10-fold higher tumor drug concentration, overcoming systemic delivery challenges (108). These innovations enhance the precision and efficacy of VASH-1-targeted interventions **Table 5**; **Figure 7**.

**Table 5:** Technological revolution in VASH-1-targeted therapy.

Technology platform	Application scenario	Core design	Breakthrough advantage	Refs
Sonoporation delivery	Tumor vascular targeting	Alternating transfection of VASH1A (pro-normalization) and VASH1B (pro-pruning) into endothelia	Spatiotemporal vascular homeostasis control	(18)
Trans-scleral sustained-release device	Choroidal neovascularization	Sustained VASH-1 release (0.31 nM/day)	Effective concentration >28 days	(107)
2’,4’-BNA-modified ASO	Systemic HCC therapy	Targets VASH2 mRNA → liver-specific accumulation (↑10× tumor concentration)	Overcomes off-target effects	(108)
Defensin-PBD-2 fusion protein	Sepsis	Activates VASH1-AKT/NF-κB pathway → targets inflammatory sites	↑60% mouse survival	(109)

## 5 Conclusion and future perspectives

VASH-1, as a critical regulatory hub within the vascular-microtubule-fibrosis network, dynamically modulates disease progression through tissue-specific functional reprogramming, showcasing substantial diagnostic and therapeutic potential. Its primary value lies in the integration of regulatory functions across various pathological processes. However, its context-dependent functional duality—manifesting as pro-tumorigenic versus anti-tumorigenic or pro-fibrotic versus anti-fibrotic effects—presents a significant translational challenge.

Future efforts should focus on three key areas: (1) Establishing standardized diagnostic protocols across diseases to address assay variability and define patient-specific biomarker thresholds; (2) Developing organ-selective delivery systems to overcome functional paradoxes and enable precise interventions; (3) Advancing AI-driven multi-omics strategies for personalized treatment prediction through deep phenotyping. With sustained innovation in engineering and rigorous clinical validation, advances in VASH-1 biology could lead to transformative therapeutic approaches for combating major human diseases.
